# Ionotropic Gelation-Based Synthesis of Chitosan-Metal Hybrid Nanoparticles Showing Combined Antimicrobial and Tissue Regenerative Activities

**DOI:** 10.3390/polym13223910

**Published:** 2021-11-12

**Authors:** Laura Lozano Chamizo, Yurena Luengo Morato, Karina Ovejero Paredes, Rafael Contreras Caceres, Marco Filice, Marzia Marciello

**Affiliations:** 1 Nanobiotechnology for Life Sciences Laboratory, Department of Chemistry in Pharmaceutical Sciences, Faculty of Pharmacy, Universidad Complutense de Madrid (UCM), Plaza Ramón y Cajal s/n, 28040 Madrid, Spain; laurloza@ucm.es (L.L.C.); yluengo@ucm.es (Y.L.M.); kovejero@ucm.es (K.O.P.); 2 Microscopy and Dynamic Imaging Unit, Fundación Centro Nacional de Investigaciones Cardiovasculares Carlos III (CNIC), Calle Melchor Fernández Almagro 3, 28029 Madrid, Spain; 3 Atrys Health, 28001 Madrid, Spain; 4 Department of Chemistry in Pharmaceutical Sciences, Faculty of Pharmacy, Universidad Complutense de Madrid (UCM), Plaza Ramón y Cajal s/n, 28040 Madrid, Spain; rafcontr@ucm.es

**Keywords:** chitosan, gold, silver, iron oxide, nanoparticles, biomedical applications, nanotechnology, antimicrobial, skin regeneration, wound healing

## Abstract

The treatment of skin wounds poses significant clinical challenges, including the risk of bacterial infection. In particular due to its antimicrobial and tissue regeneration abilities chitosan (a polymeric biomaterial obtained by the deacetylation of chitin) has received extensive attention for its effectiveness in promoting skin wound repair. On the other hand, due to their intrinsic characteristics, metal nanoparticles (e.g., silver (Ag), gold (Au) or iron oxide (Fe_3_O_4_)) have demonstrated therapeutic properties potentially useful in the field of skin care. Therefore, the combination of these two promising materials (chitosan plus metal oxide NPs) could permit the achievement of a promising nanohybrid with enhanced properties that could be applied in advanced skin treatment. In this work, we have optimized the synthesis protocol of chitosan/metal hybrid nanoparticles by means of a straightforward synthetic method, ionotropic gelation, which presents a wide set of advantages. The synthesized hybrid NPs have undergone to a full physicochemical characterization. After that, the in vitro antibacterial and tissue regenerative activities of the achieved hybrids have been assessed in comparison to their individual constituent. As result, we have demonstrated the synergistic antibacterial plus the tissue regeneration enhancement of these nanohybrids as a consequence of the fusion between chitosan and metallic nanoparticles, especially in the case of chitosan/Fe_3_O_4_ hybrid nanoparticles.

## 1. Introduction

In the last decades, thanks to huge advances in nanotechnology, nanoparticles (NPs) have been widely studied for their application in biomedicine [1]. These NPs can be organic, inorganic or hybrid NPs, each of them offering different advantages and providing a wide range of applications in numerous fields [2,3,4,5,6,7,8]. Nowadays, the attention to hybrid NPs is increasing because they are more versatile and effective in overcoming obstacles compared to inorganic and/or organic ones alone [9,10,11]. The combination of inorganic and organic materials can lead to a synergistic effect or to a new hybrid nanosystem with unique properties [4,11,12]. These particles are being studied as carriers for drug delivery, gene therapy, imaging, theranosis, tissue regeneration, vaccines, among other uses [9].

In the context of tissue regeneration, skin cell regrowth is an interesting field considering that the integrity of healthy skin is crucial for maintaining human body’s physiological homeostasis [13,14]. Skin is the largest organ of human body, and thus highly vulnerable to a variety of damages and lesions, such as chronic wounds and deep burns of difficult healing [13]. These can affect millions of persons worldwide and can lead to serious clinical complications associated with long term hospitalization and expensive treatments without complete recovery of healthy skin conditions. Furthermore, many cutaneous injuries are followed by bacterial infection that can aggravate and slow down the skin regeneration process. Recent studies about tissue regeneration together with recent advances in the biomaterials and nanotechnology field, have been providing interesting and promising alternatives to the current treatments, offering the possibility of restoring the skin with its intrinsic functions [15,16,17,18]. Thus, in order to treat skin lesions, it is necessary to achieve wound closure and skin regeneration while avoiding bacterial infections [19]. To this aim, nanomedicine provides interesting and effective new tools to achieve this purpose [1,19]. Specifically, chitosan (CS)-based nanoparticles (NPs) have been revealed to be promising candidates for skin damage treatment [20].

Chitosan (CS) is a natural polysaccharide that shows interesting intrinsic properties such as biocompatibility, biodegradability, no antigenicity, mucoadhesive behaviour, anti-inflammatory and antibacterial abilities, making it an attractive material for biomedical applications [21,22,23]. Furthermore, recently, its ability to promote and accelerate tissue repair and regeneration processes was investigated [24].

On the other hand, inorganic NPs such as silver (Ag), gold (Au) or iron oxide (Fe_3_O_4_) NPs, have demonstrated promising properties related with tissue regeneration or wound healing, in addition to intrinsic therapeutic properties and the possibility of surface functionalization, useful to attach drugs or biomolecules for drug targeting [25].

Among different metal oxide nanoparticles, Fe_3_O_4_ NPs are well-known in the biomedical field because of their unique superparamagnetic properties and their different surface chemistry and functionalization. These were intensively applied for cell separation, tissue repair, targeted drug delivery (by using a magnet), bio-labelling material, hyperthermia and MRI contrast agents [2,26,27,28,29,30,31]. Furthermore, previous studies have reported that topically applied iron chelators and their novel therapeutic agents are able to improve the wound healing [32]. Recently, the antibacterial properties of these NPs have been studied in some depth [33,34], such as their application in regenerative medicine and tissue engineering [35], above all for neural and musculoskeletal tissues [36]. Thus, an interesting approach would be the incorporation of Fe_3_O_4_ NPs within CS polymeric NPs in order to combine their individual regenerative and antimicrobial properties in one unique element. In this sense, some examples of these synergistic effects have been reported although, in the vast majority of cases, only one bioactive property (antimicrobial or tissue regenerative) has been evaluated separately [37].

Ag NPs have demonstrated great potential in different biological applications, such as diagnosis, drug delivery, novel antimicrobial agents and regeneration materials [38,39,40]. In recent years, the antimicrobial behaviour of Ag NPs has led to increased demand for their applications in the biomedical field, including wound dressings, artificial implantation, and as antitumor drug carriers [41,42]. These NPs were also used as coatings for implantable medical devices, preventing infection and promoting wound healing, antibiotic delivery, microbial diagnostics, and antibacterial vaccines to control bacterial infections [43]. The main problem of these particles is associated to their cytotoxicity. Thus, a promising approach could be represented by the incorporation of Ag NPs into CS film matrices to minimize the severe toxicity of Ag NPs while maintaining or enhancing the antimicrobial effects [44,45,46,47].

Because of their unique surface, electronic and optical properties, Au NPs have been also widely used in biomedicine in areas such as biosensing, gene transfer and drug transport [11,48,49]. In the recent years, as a result of the nano-bio interaction with skin lipids, Au NPs have been shown to be capable of opening the stratum corneum and penetrating the skin barrier, so that their combination with CS would facilitate and improve their biological effects [50,51].

Therefore, the encapsulation of these inorganic NPs inside CS appears as an interesting and useful approach to investigate possible synergic effects between skin regeneration and wound healing while reducing eventual toxic side effects related to their nature.

To synthesize these hybrid NPs, different strategies have been described in literature [52]. However, the great majority of them require toxic crosslinkers (i.e., glutaraldehyde) and complex and not scalable strategies based on emulsification (with the use of harmful and organic solvents), reverse micelles (microemulsions) or phase inversion precipitation techniques among others [52,53,54]. In turn, little literature describing the application of the ionotropic gelation strategy for the synthesis of chitosan plus metal NPs hybrids has been published to date. Differently from the majority of reported strategies, this cheap, green and fully scalable method is based on a finely tuned and quick ionic crosslinking that involves the positive charges of chitosan and the negative charges of tripolyphosphate (TPP) in aqueous medium [55].

In this work, an accurate designed synthesis of CS-based hybrid NPs based on this green and simple synthetic strategy followed by an accurate physicochemical characterization will be described. As a result, a standard protocol based on an ionotropic gelation strategy for the preparation of hybrid CS/Fe_3_O_4_, CS/Ag and CS/Au NPs will be successfully optimized. After their physicochemical characterization, the biocompatibility, antimicrobial activity and tissue regeneration capability of the products will be studied and characterized for each hybrid.

## 2. Materials and Methods

### 2.1. Reagents

All the reagents used to synthesize polymeric and inorganic nanoparticles (NPs) were purchased from Merck (Darmstadt, Germany). These are: iron (III) chloride hexahydrate (FeCl_3_·6H_2_O), iron (II) chloride hydrate (FeCl_2_·6H_2_O), ammonium hydroxide (NH_4_OH) and nitric acid (HNO_3_) for iron oxide (Fe_3_O_4_) NPs, dodecylbenzene sulfonic acid (DBSA), aniline, silver nitrate (AgNO_3_, ≥99%) for silver (Ag) NPs, HAuCl_4_·3H_2_O and trisodium citrate dehydrate for gold (Au) NPs, and chitosan (CS) (low molecular weight, 50–290 kDa and deacetylation degree of 75–85%) and sodium triphosphate pentabasic (TPP, ≥98%) for CS NPs synthesis. For cell culture and cell viability assays, Dulbecco’s Modified Eagle’s medium (DMEM) was obtained by Gibco (Waltham, MA, USA). Dimethylthiazolyl-diphenyl-tetrazolium bromide (MTT), dimethyl sulfoxide (DMSO) and FBS were bought by Merck. Finally, non-essential amino acids (NEAA) and sodium pyruvate were obtained from Hyclone (South Logan, UT, USA) while penicillin/streptomycin from Lonza (Basel, Switzerland).

### 2.2. Nanoparticles Synthesis

#### 2.2.1. Iron Oxide Nanoparticles

Fe_3_O_4_ NPs were prepared following the Massart co-precipitation protocol with some modifications to control the particle size to 8 nm and to reduce the size distribution below a polydispersity degree of 0.2 (standard deviation/mean size) [56,57,58].

Briefly, magnetite (Fe_3_O_4_) NPs were prepared by fast co-precipitation (40 mL/s) of 425 mL of a mixture of FeCl_3_·6H_2_O (0.09 mol) and FeCl_2_·6H_2_O (0.054 mol) in 75 mL of alkaline medium (NH_4_OH, 25%). After the synthesis, the particles were washed three times with distilled water in the presence of a permanent magnet. Then, an acid treatment was carried out, based on the oxidation of magnetite to maghemite (γ-Fe_2_O_3_), which is more stable in time and suitable for biomedical applications. 300 mL of HNO_3_ (2 M) were added to the particles and stirred during 15 min. Then, the acid was removed by magnetic decantation, and 75 mL of Fe(NO_3_)_3_ (1 M) and 130 mL of water were added. The mixture was heated up to boiling temperature and stirred for 30 min. NPs were cooled to room temperature and after magnetic decantation (process in which a magnet is used to attract all magnetic components allowing their separation from a mixture), the supernatant was substituted by 300 mL of HNO_3_ (2 M) and stirred for 15 min. Finally, the particles were washed three times with acetone and redispersed in water, and a rotary evaporator was used to remove any acetone waste and concentrate the sample.

#### 2.2.2. Silver Nanoparticles

Ag NPs were prepared following a previous described protocol [59]. Briefly, 3.48 g of DBSA were dissolved in 90 mL of distilled water, followed by the addition of 181 µL of aniline (186 mg) and vigorous stirring at 500 rpm until the solution took on a transparent, yellowish appearance. Then, 10 mL of a 0.2 M AgNO_3_ aqueous solution were added and the resultant mixture was stirred for 15 min at 500 rpm. Next, this solution was heated to 90 °C in an oil bath and, when the temperature was reached, 4.5 mL of 3M NaOH aqueous solution were added. The mixture was kept at 90 °C for 1 h until a white AgCl precipitate was not observed when a 1 M NaCl solution was added to the reaction system, which implied almost the 100% yield reaction. The final colloidal dispersion was left to cool-down to room temperature and then was washed twice by centrifugation at 7500 rpm and 15 °C for 20 min. Then, the final Ag colloids were re-dispersed in distilled water.

#### 2.2.3. Gold Nanoparticles

Au@citrate NPs were prepared following the Turkevich precipitation protocol in water [60]. Briefly, 95 mL of an aqueous solution containing 0.45 mM HAuCl_4_·3H_2_O was heated to boiling. Then, 5 mL of aqueous trisodium citrate solution (1g/100 L) was added under vigorous magnetic stirring. The initial yellowish solution changed immediately from colorless to black solution, and finally to a red wine color. Then, this colloidal dispersion was left to cool-down until room temperature and one part was washed by centrifugation at 6500 rpm and 15 °C for 5 min. Then, the final AuNPs colloids were re-dispersed in water (Au@citrate). The final Au concentration was calculated by the absorbance at 400 nm and confirmed by the inductively coupled plasma (ICP) technique.

#### 2.2.4. Chitosan Nanoparticles

CS NPs were synthesized using the ionotropic gelation of CS with TPP method [61,62,63,64]. Briefly, CS was dissolved in an acid aqueous solution of 0.1% (*v/v*) acetic acid obtaining a dissolution of 1 mg/mL. Then, this solution was filtered by 0.45 µm using sterile filters and the pH was adjusted to 4.7–4.8. TPP was dissolved in distilled water in a concentration of 5 mg/mL and filtered by 0.22 µm using sterile filters. NPs were spontaneously formed by the incorporation of 1.2 mL of TPP solution in 3 mL of CS solution, under mild magnetic stirring at room temperature during 30 min. The prepared CS/TPP ratio resulted between 2.4/1 and 2.6/1 (*w/w*). To wash the synthesized NPs, the sample was centrifuged on a 4 µL of glycerol bed at 7000 rpm, 15 °C for 30 min, and resuspended in 100 µL of filtered distilled water after having discarded the supernatant.

#### 2.2.5. Hybrid Nanoparticles

Hybrid CS-based NPs (CS/Fe_3_O_4_ NPs, CS/Ag NPs and CS/Au NPs) were synthesized following a similar previously described protocol [55]. Briefly, 1.2 mL of TPP solution contained metal NPs in a concentration of 0.2 mg/mL were added to 1 mg/mL CS solution. The final CS/TPP ratio resulted in 2.6/1 (*w/w*) for the three hybrid samples. To wash the synthesized NPs, the sample was centrifuged on a 4 µL of glycerol bed at 7000 rpm, 15 °C for 30 min, and resuspended in 100 µL of filtered distilled water after having discarded the supernatant.

### 2.3. Nanoparticles Morphology, Size and Surface Zeta Potential Characterization

First, the morphology of all the synthesized NPs (metal, CS and hybrid NPs) was examined by transmission electron microscopy (TEM JEM 1400, JEOL, Akishima, Tokyo, Japan). Aqueous suspensions of the inorganic NPs were placed on 200 mesh copper grids coated with formvar/carbon without any staining. Colloidal suspensions of CS and hybrid samples were placed on the same grids after staining with 2% (*w/v*) of phosphotungstic acid. The core size of each sample was achieved by using ImageJ program to measure the longer side of more than 200 nanoparticles. After that, all the values were processed by Origin obtaining the mean core size distribution (log-normal fit).

First, the morphology of all the synthesized NPs (metal, CS and hybrid NPs) was examined by transmission electron microscopy (TEM JEM 1400, JEOL, Akishima, Tokyo, Japan). Aqueous suspensions of the inorganic NPs were placed on 200 mesh copper grids coated with formvar/carbon without any staining. Colloidal suspensions of CS and hybrid samples were placed on the same grids after staining with 2% (*w/v*) of phosphotungstic acid. The core size of each sample was achieved by using ImageJ program to measure the longer side of more than 200 nanoparticles. After that, all the values were processed by Origin obtaining the mean core size distribution (log-normal fit).

Measurements of NPs size and surface zeta potential were performed on freshly prepared and purified samples using a DLS Zetasizer Nano Zen 3600 (Malvern Instruments, Malvern, UK). For the particle size analysis, each sample was diluted to the appropriate concentration with filtered distilled water. And for the zeta potential analysis, samples were diluted in potassium nitrate (KNO_3_, 0.01 M).

### 2.4. Fourier Transform Infrared (FTIR) Spectroscopy

FTIR measurements were carried out with lyophilized samples and were recorded using a Nicolet iS50 instrument (Thermo Fisher Scientific, Waltham, MA, USA) equipped with a KBr beam-splitter of. The FTIR spectra were used to verify the formation of hybrid NPs.

### 2.5. Determination of Nanoparticles Synthesis Yield

The CS and hybrid NPs synthesis yield was determined by gravimetry. For this purpose, fixed volumes of NPs suspension were centrifuged at 7000 rpm, 15 °C for 30 min and, after that the supernatant was discarded, the pellets were lyophilized during 24 h using a LyoQuest (Telstar, Terrassa, Barcelona, Spain) lyophilizer and subsequently weighed. Three batches for each formulation were analysed.

### 2.6. Determination of Inorganic Nanoparticles Encapsulation

To determine the encapsulation grade of inorganic NPs in hybrid NPs, samples were centrifuged at 7000 rpm, 15 °C for 30 min and the supernatants and the pellets were separated for different analysis. First, pellets were lyophilized over 24 h using a using a LyoQuest (Telstar) lyophilizer and then analyzed by gravimetry. In addition, both pellets and corresponding supernatants were analyzed by the inductively coupled plasma (ICP, SPECTRO Arcos, SPECTRO, Kleve, Germany) technique to determine the concentration of metal NPs and, therefore, their encapsulation yield in the CS NPs.

### 2.7. Inorganic Nanoparticles Quantification

To determine Fe, Ag and Au amount in inorganic NPs, ICP analysis was used (as described in Section 2.6). Furthermore, Au and Fe concentrations were also measured by spectrophotometric methods. In more details, Au was determined measuring the absorbance at 400 nm. At this wavelength the main contribution to absorbance comes from absorption related to interband transitions in metallic gold which would thus be used to determine the amount of gold, regardless of particle size and shape. It has been reported that a value of 1.2 for the absorbance at 400 nm corresponds to an Au^0^ concentration of 0.5 mM [65].

For iron determination a Spectroquant Iron Test (Merck, Darmstadt, Germany) was used following the provider’s instructions. All iron ions are reduced to iron (II) ions. In a thioglycolate-buffered medium these react with a triazine derivative to form a red-violet complex that is determined photometrically. Briefly, 40 ul of sample was digested with 40 µL of H_2_O_2_/HNO_3_ (1:1.4) during 1 h at 60 °C. After digestion, the volume was adjusted to 5 mL with distilled water and 3 drops of kit reagent Fe-1 was added. This mixture was incubated for 3 min and then the absorbance was measured at 565 nm.

### 2.8. Cell Viability Assay

Mouse embryonic fibroblasts (MEF) were used to perform the cell viability assay, in order to study the toxicity of all the synthesized nanoparticles. This cell line was grown and maintained in DMEM supplemented with 10% FBS, 1% NEAA, 1% sodium pyruvate 100 mM and 1% penicillin/streptomycin. This culture was maintained at 37 °C and in an atmosphere with 5% CO_2_. Samples were redispersed in culture media, without phenol red, at different concentrations, and always sonicating repeatedly before cellular incubation.

Cells were seeded (4000 per well) in a 96-well culture plate and grown for 48 h at 37 °C. Then, cells were incubated for 24 h at 37 °C with different concentrations of each sample. After that, all the solutions were discarded and replaced by 100 µL of medium (without FBS and phenol red) and 10 µL of a 12 mM MTT solution in medium in each well. The reaction was incubated for 3 h, and then 85 µL of supernatants were removed, and 100 µL of DMSO were added to dissolve the formazan, leaving it 15 min in gentle agitation. Cell viability was estimated by measuring absorbance at 570 nm, knowing that the higher the absorbance, the higher the viability.

### 2.9. Antibacterial Activity Test

Antibacterial tests were carried out with bacteria in suspension (in planktonic state) with a single concentration of the different NPs samples. Briefly, 0.5 mL of bacterial suspension containing 2·10^6^ bacteria/mL of *Escherichia coli* in PBS 1X was placed in a 24-well culture plate together with 0.5 mL of the different NPs samples, in duplicate. This suspension was incubated for 4 h at 37 °C and continuous shaking. After this, 20 µL of a 1/1000 dilution from each well were seeded onto tryptic soy agar plates and incubated overnight at 37 °C. The next day, the colony formation on the different plates was checked.

### 2.10. Wound Healing Assay

MEF cells were seeded (35,000 per well) in a 24-well culture plate for 48 h, until a cell monolayer was created. The monolayer was scratched with a pipette tip and the medium was absorbed with vacuum. Then, the cells were incubated with the different samples (inorganic, CS and hybrid NPs) at different concentrations and the migration into the gap was imaged at 0, 24 and 48 h. Images were analysed in ImageJ (V 1.8.0, https://imagej.nih.gov/ij/) using the Wound Healing Tool.

## 3. Results and Discussion

### 3.1. Nanoparticles Preparation and Characterization

**Figure 1:**
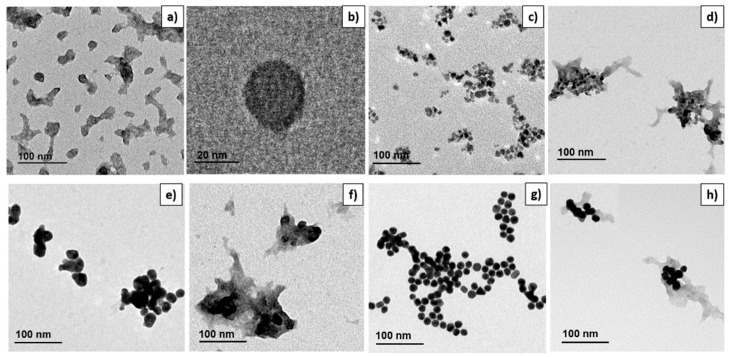
TEM micrographs of (**a**) CS NPs, (**b**) higher magnification of CS NPs, (**c**) Fe_3_O_4_ NPs, (**d**) hybrid CS/Fe_3_O_4_ NPs, (**e**) Ag NPs, (**f**) hybrid CS/Ag NPs, (**g**) Au NPS and (**h**) hybrid CS/Au NPs.

As first step, the metallic Ag, Au and Fe_3_O_4_ NPs were synthesized as described in the Material and Methods section, finally showing a core size of 33.02 nm (± 13.02 nm), 14.05 nm (± 1.61 nm) and 8.18 nm (± 1.57 nm), respectively, as measured by TEM microscopy (Figure 1c,e,g and Figure S1; Table 1). When dispersed in aqueous medium, all the NPs tended to form small aggregates displaying hydrodynamic diameters of about 100 nm, 30 nm and 20 nm, respectively (analysed by the DLS technique, Table 1). In general, the metallic nanoparticles appear as spherical and monodisperse, except for Ag NPs that show a slightly higher polydispersity index (PDI = 0.29, Table 1).

**Scheme 1:**
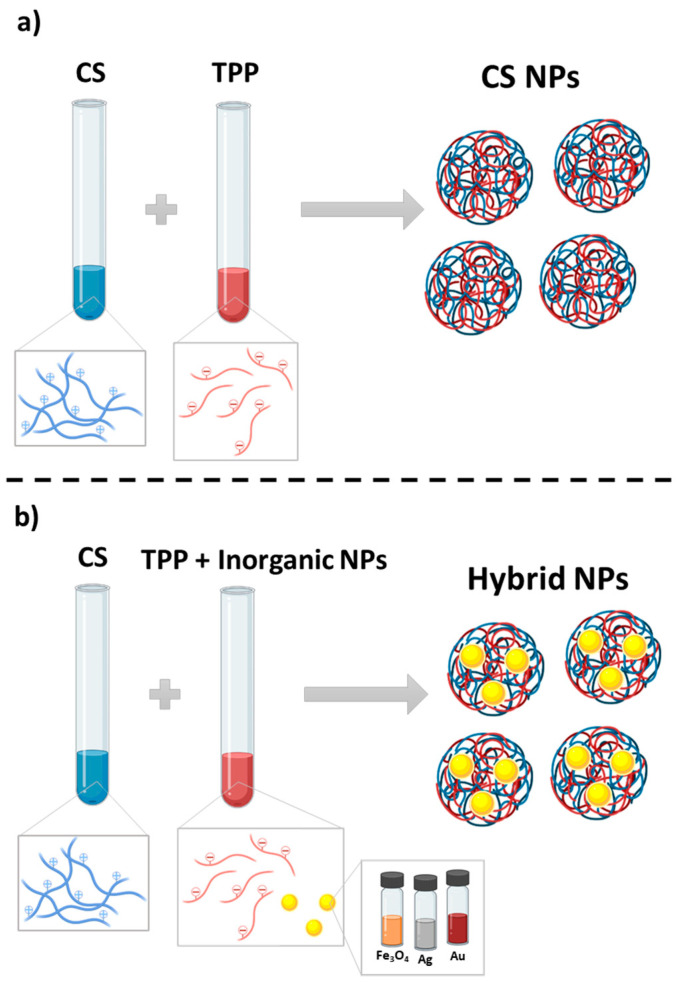
General scheme for the synthesis of (**a**) CS NPs and (**b**) hybrid CS/Metal NPs. Created with BioRender.com.

After that, CS and hybrid NPs were prepared using the ionotropic gelation technique (Scheme 1a,b, respectively) [61]. As key parameter, various ratios between the constituents CS and TPP and/or inorganic NPs were studied to find the best relation for obtaining both the highest CS NPs yield and inorganic NPs encapsulation (Table 2). These conditions are related to polymer concentration. In fact, lower CS concentrations led to smaller size and higher yield due to mechanism of hybrid NPs formation [61]. Thanks to their amino groups that are positively charged at acid pH, CS chains are able to form an ionic network because of the crosslinking ability of negatively charged TPP [66] (Scheme 1).

Hence, the highest yield (65%, Table 2) of CS NPs was obtained using a CS/TPP ratio of 2.4/1 (*w/w*). These NPs showed a spherical shape with a TEM core size of 34 nm (Figure 1a,b and Figure S2). In aqueous medium, the CS NPs tend to form small aggregates with a hydrodynamic size around 100 nm (analysed by DLS technique) (Table 1). From the TEM micrographs in Figure 1a it is possible to observe that these CS NPs tend to form enlarged aggregates with an irregular necklace-shape. The aspect of these aggregated is in agreement with data reported in literature and could be correlated with similar sample-drying procedure, similar polymer molecular weight (MW) and similar deacetylation (DA) degree [67,68]. In fact, it was reported that bare CS NPs prepared by ionotropic gelation strategy and using CS with similar physical properties (MW and DA), showed the same shape (spherical) and the similar tendency to form similar necklace-shaped aggregates [68,69,70,71,72,73,74].

To promote their encapsulation within the polymeric matrix, the inorganic NPs were incorporated during the synthesis of CS NPs by means of their previous dispersion into the TPP solution. After testing different CS/TPP and TPP/inorganic NPs ratios (*w/w*), hybrid CS based NPs, with a hydrodynamic size of about 120 nm in all cases, were successfully prepared (Figure 1d,f,h; Table 1).

TEM micrographs show inorganic NPs inside CS NPs, confirming the successful encapsulation. These hybrid NPs are forming small aggregates with similar necklace shape to the CS ones. Nevertheless, the presence of some organic CS NPs (without encapsulated inorganic NPs), can be also appreciated demonstrating a no uniform distribution of inorganic NPs inside the CS. This behavior is mainly appreciable for Ag/CS and Au/CS NPs, probably due to the higher inorganic NPs core size than Fe_3_O_4_ NPs and to the inorganic NPs concentration used in the synthesis.

By applying the optimized conditions, the encapsulation of metal NPs was almost quantitative in all cases (Table 2). However, the overall yields of hybrid NPs were reduced with respect to bare CS NPs (from 65% to about 30%) (Table 2).

To confirm the successful formation of hybrid NPs as well as to quantify inorganic elements presence, surface ζ-potential, FT-IR and inductive coupled plasma (ICP) analyses were carried out.

At pH 7, the surface potential of hybrid NPs, was greatly increased from negative or near-to-zero values (−11.8 mV, −17.4 mV and 3.6 mV for Ag, Au and Fe_3_O_4_ NPs, respectively) to about +7.5 mV for all hybrid-based CS NP, demonstrating the successful inclusion of the metallic NPs within CS NPs (Table 2). In fact, the more positive ζ-potential is related to the presence of positive charged CS on hybrid NPs surface that finally masks the original negative charges. On the other hand, at the same pH value, the surface ζ-potential of CS NPs slightly decreased from 8.1 up to 7.5 mV as a consequence of the entrapment of the more negative metallic NPs within its structure (Table 2).

**Figure 2:**
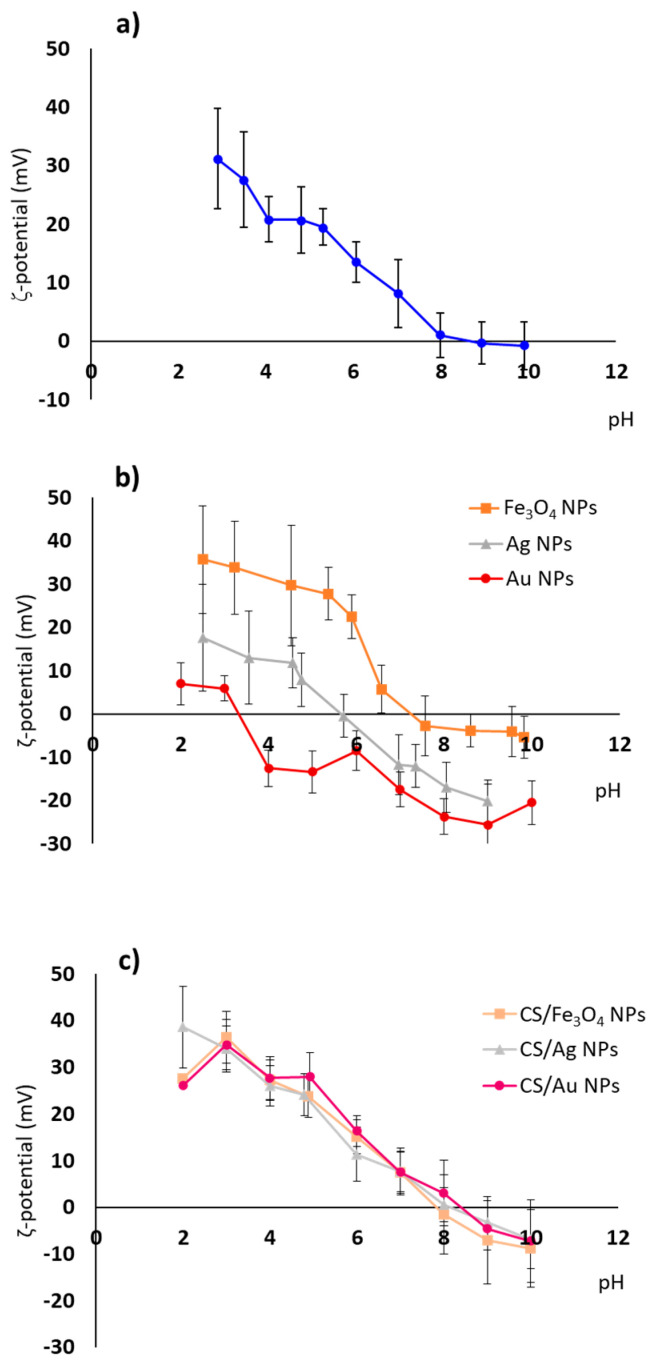
Surface ζ-Potential graphics of (**a**) CS NPs, (**b**) inorganic NPs and (**c**) hybrid CS-based NPs.

The isoelectric point of all the particles was also measured (Figure 2) and resulted almost the same (pH 8, approximatively, Figure 2c) for hybrid CS/metal NPs.

**Figure 3:**
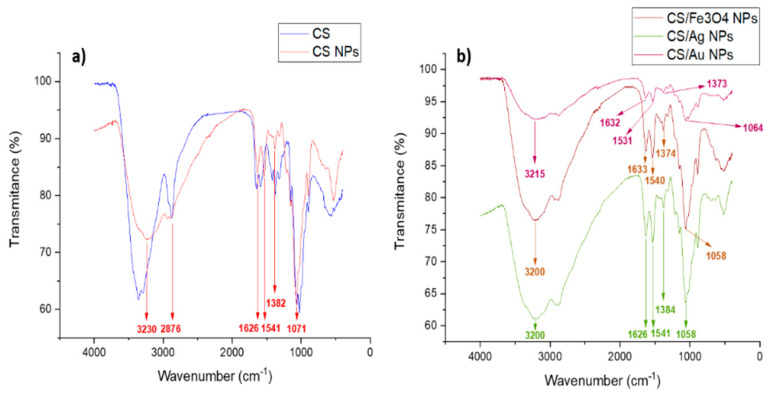
FT-IR spectra of (**a**) CS NPs compared to CS polymer and (**b**) hybrid CS based NPs.

The FTIR spectra of CS and hybrid CS based NPs were recorded (Figure 3). The spectra of the polymer (CS) and each inorganic NP are reported in Supplementary Materials.

More in details, Figure 3a shows the spectrum of CS NPs compared with the polymer alone. Pristine CS shows a band at 3363 cm^−1^ that was assigned to O–H and N–H group stretching vibrations, while the band at 2876 cm^−1^ was due to the stretching vibrations of the aliphatic C–H bonds. The signals at 1642 cm^−1^ and 1583 cm^−1^ correspond to the stretching vibration of the amide bonds I, II and III. And the bands at 1377 cm^−1^ and 1024 cm^−1^ was assigned to C–N and C–O–C bonds, respectively (see Supplementary Materials). The peaks attributed to amide bonds shifted decreasing to 1626 cm^−1^ and 1541 cm^−1^, which could be caused by the interaction between NH_3_^+^ groups of chitosan and phosphate groups of TPP [75]. The peak corresponding to O–H and N–H groups moved to 3230 cm^−1^.

In Figure 3b, the FTIR spectra of hybrid NPs is reported. In CS/Ag NPs, the same peaks reported in CS NPs spectrum can be found with exception of the band at 3230 cm^−1^ of the O–H and N–H groups that decreased to 3200 cm^−1^, suggesting the chelation of Ag with both amino and hydroxyl groups of chitosan. The same typical pattern can be observed in CS/Fe_3_O_4_ and CS/Au NPs.

Thanks to the ICP-OES analysis, the amount of each metallic NP encapsulated within the CS polymeric matrix in each hybrid NPs was determined (Table 2). In more details, 0.26, 0.16 and 0.14 mg of Fe_3_O_4_, AgNP and AuNPs, respectively, were included in 1 mg of each hybrid CS NPs. Altogether, all the reported characterizations confirmed the successful preparation of hybrid CS/metal NPs.

### 3.2. Cytotoxicity Tests

After a deep physicochemical characterization of all the synthesized NPs (inorganic, CS and hybrid NPs), with the aim of using these NPs in biomedical field, firstly their biocompatibility was tested in vitro. Fibroblasts cells (MEF) were chosen with the objective of testing all these nanomaterials as wound healing and skin regeneration tools. These cells have been selected for our studies as they show a pivotal role in the skin wound healing setting [76]. Thus, the demonstration of a potential proliferative activity with a concomitant moderate toxicity profile of our CS-based hybrid nanosystem on these cells could represent a very promising translational model.

**Figure 4:**
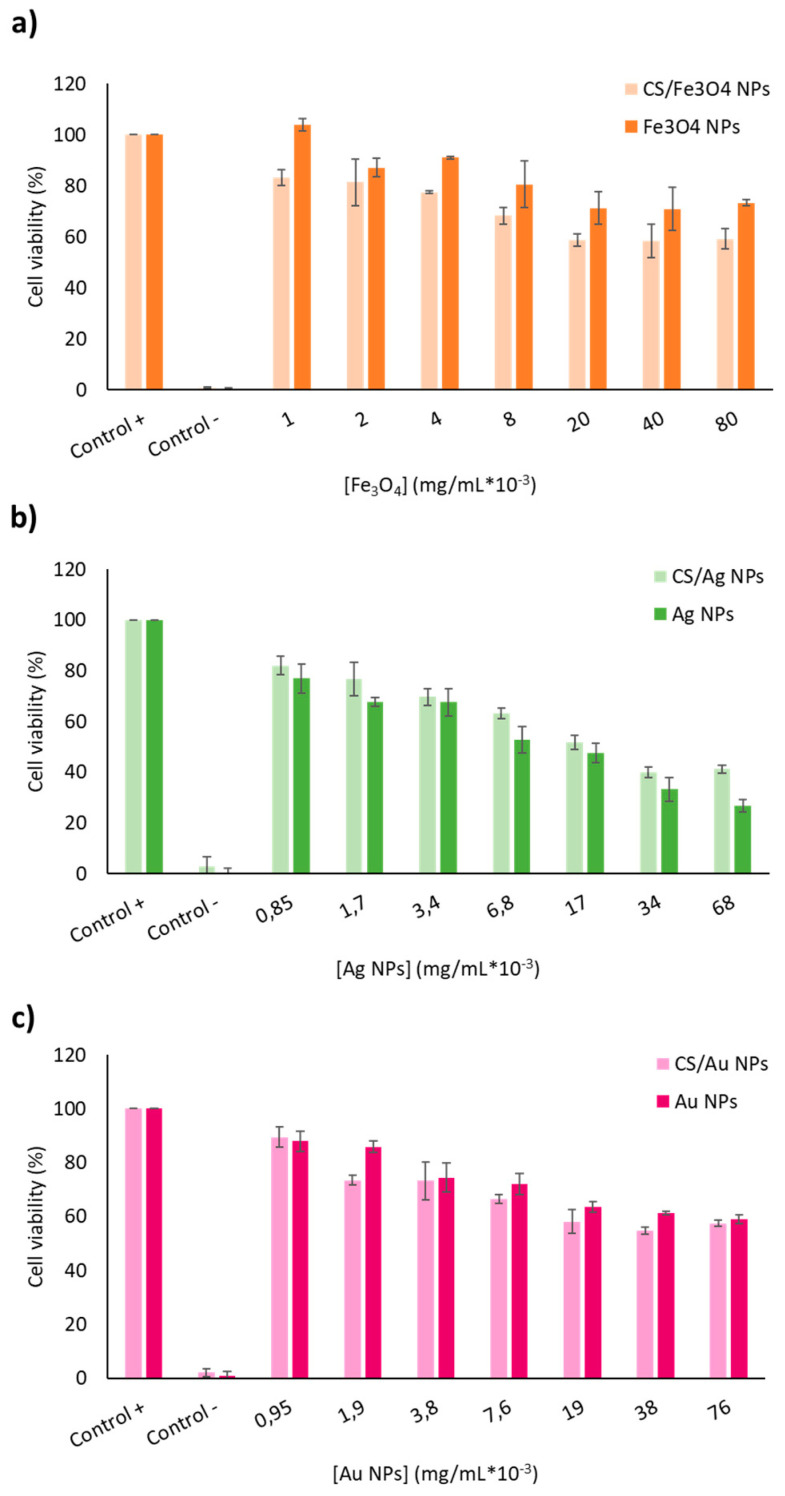
Cell viability assay of fibroblast as a function of different concentration of hybrid CS/metal nanoparticles versus free metal nanoparticles at the same concentration. (**a**) CS/Fe_3_O_4_ NPs versus free Fe_3_O_4_ NPs. (**b**) CS/Ag NPs versus free Ag NPs. (**c**) CS/Au NPs versus free Au NPs.

In a first set of experiments, the potential cytotoxicity of all NPs on MEF cell line was assessed at different samples concentrations by using the MTT cell viability assay. As expected, CS NPs resulted non-toxic up to concentrations of 0.5 mg/mL being the final MEF availability at this concentration more than 75% (Figure S3). In turn, in comparison with CS NPs, inorganic NPs showed higher cytotoxicity both in their free and encapsulated forms (Figure 4). Fe_3_O_4_ NPs showed a behaviour similar to bare CS NPs, but up to a concentration of 0.1 mg/mL; at 0.25 mg/mL the cells’ cytotoxicity increased. Biocompatibility of Au NPs was reduced to 60% at the concentration of 0.025 mg/mL while Ag NPs, at 0.0125 mg/mL, produced a 50% toxicity, showing the highest cytotoxicity values. Despite the toxicity of metal nanoparticles [77] and especially in the case of more toxic silver NPs, the incorporation of these inorganic NPs into CS network reduced their cytotoxicity, due to the presence of the most biocompatible CS layer on NPs surface. CS/Fe_3_O_4_ NPs at a concentration of 0.08 mg/mL of Fe_3_O_4_ NPs showed a viability in cells of around 60%, having the same cell viability in the case of CS/Au NPs, at a concentration of 0.076 mg/mL of Ag NPs. These viabilities were almost the same in comparison with inorganic NPs alone. Nonetheless, the cell viability dropped up to 26% with a concentration of 0.068 mg/mL of free Ag NPs, while the cell viability was preserved at 41% at the same concentration of Ag NPs but encapsulated within the CS polymeric matrix, therefore, demonstrating the reduction of their cytotoxicity once in their CS-based hybrid form (Figure 4).

### 3.3. Antibacterial Activity Test

In order to assess the potential ability of these hybrid NPs to inhibit the bacterial growth, preliminary antibacterial tests were carried out on Gram negative *E. coli* as model. In fact, *Escherichia coli* strains frequently are isolated from skin and soft tissue infections [78]. To assess the antibacterial ability of CS NPs without impacting on its possible therapeutic ability, the highest no toxic concentration of CS NPs on MEF was tested (0.5 mg/mL). The same concentration of CS metal hybrid nanoparticles was applied in each case, and the relative amount of free metal NPs contained in each hybrid (calculated by ICP, Table 2) was used as control (see Materials and Methods).

**Figure 5:**
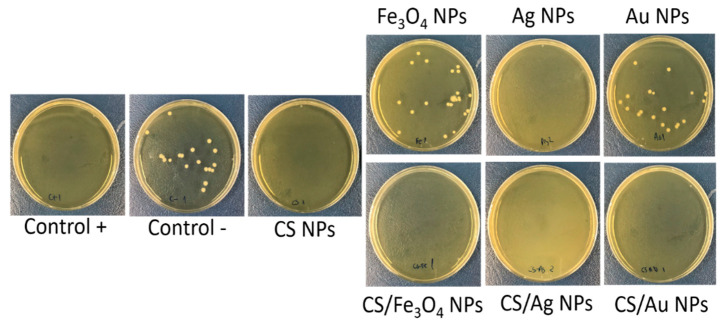
Antibacterial activity of CS based hybrid NPs compared to respective inorganic NPs measured by their ability to inhibit E. coli colonies formation.

The results showed that bare CS NPs exhibited good antimicrobial properties as no *E. coli* colonies were formed on the plate upon incubation with the polymeric nanodrug (Figure 5). As expected, after incubation with free metallic nanoparticles, only AgNPs showed inhibitory activity while Fe_3_O_4_ NPs and AuNPs did not exert any antimicrobial activity as demonstrated by the *E. coli* colonies formation (Figure 5). In contrast, both inactive NPs (Fe_3_O_4_ and Au) turned in antimicrobial ones upon their encapsulation within the chitosan matrix. In fact, at the same metal concentration tested in their free form, both hybrids showed complete growth inhibition of *E. coli* colonies, therefore, confirming the raise of their antimicrobial ability (Figure 5) upon their hybridization with CS polymer. Altogether, these results clearly demonstrate the capability of CS/metal nanohybrids to inhibit bacterial growth thanks to presence of chitosan.

### 3.4. Wound Healing Assay

**Figure 6:**
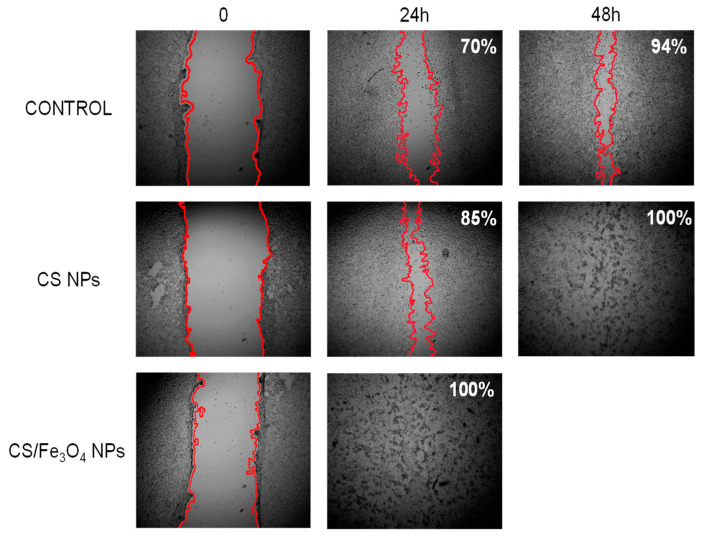
Hybrid NPs accelerated migration of fibroblast in scratch assay. Positive control at 0 h, 24 h and 48 h versus CS NPs (33.75 μg/mL) at 24 h and 48 h versus CS/Fe_3_O_4_ NPs (33.75 μg/mL) at 24 h. The red lines indicate the fronts of migrating cells, and the percentage indicates the healed area.

Beside the antibacterial ability of our CS metal hybrid nanoparticles, we have also evaluated their potential capability to also promote, at the same time, skin wound healing [79]. Toward this end, we have carried out a scratch assay consisting in culturing MEF fibroblast in Petri dishes, generating a standard wound and assessing the ability of fibroblasts to heal that wound (in form of cell migration) in absence and in presence of each nanoparticle as well as of their individual constituent (Figure 6 and Figure S4). The extent of cells’ migration inside the scratch was measured by imaging analysis after 24 h and 48 h of incubation with the different NPs at different concentrations and comparing the residual wound area vs. the initial one. Upon analysis of all images recorded at all times and for each NPs at all concentrations studied, as general trend, it results clear that CS NPs and hybrid CS metal NPs promoted a faster wound healing process in comparison to the positive control (Figure S4). In more details, CS NPs at 33.75 μg/mL promoted 85% of scratch healing at 24 h and a quantitative healing at 48 h being 70% and 94% the healed areas at 24 h and 48 h, respectively, for positive control (Figure 6). Interestingly, at the same concentration, the CS/Fe_3_O_4_ NPs promoted the fastest cells migration with a quantitative wound healing after only 24 h (Figure 6). In the case of CS/Ag and CS/Au NPs, the appearance of this cell migration promotion activity was not appreciated (Figure S4). Therefore, besides the generation of antimicrobial activity, the hybridization between CS and Fe_3_O_4_ metal nanoparticles is able to also induce a tissue regenerative ability.

## 4. Conclusions

In sum, in this work we have investigated the generation of a multifunctional hybrid nanoplatform composed by metallic nanoparticles embedded within a chitosan matrix. The goal of our strategy was to achieve a nanocomposite showing at the same time tissue regenerative and antimicrobial properties for its application in wounded skin management. The synthesis of the chitosan/metal hybrid nanoparticles has been carried out by means of the ionotropic gelation strategy. This green and practical synthetic protocol is characterized by its flexibility and scalability, thus, being a great added value toward its translational biomedical application. The achieved nanoparticles have been synthesised in good overall yield as a function of the ratio between the chitosan polymer, the TPP crosslinker and the amount of the metallic nanoparticles. The successful preparation of the nanohybrids has been confirmed in depth by different physico-chemical techniques (TEM, DLS, FTIR and ICP). After the physicochemical characterization, the biomedical activity of the bare CS NPs as well as of their hybrids with embedded metal nanoparticles was assessed. In general, all the CS-based nanoparticles showed good biocompatibility being the bare CS-NPs the most biocompatible ones. As a result of the entrapment of metal nanoparticles within the chitosan matrix, the CS metal hybrid nanoparticles acqured a good antibacterial activity. In fact, apart from AgNPs, the bare metal NPs did not show any initial antimicrobial activity. In addition to the antimicrobial activity, the combination between CS nanoparticles and Fe_3_O_4_ metal NPs metal hybrid also showed a good tissue regenerative activity as they promoted the fastest (24 h) and quantitative wound healing in fibroblast scratch model in comparison with the bare CS NPs and the CS Au and Ag hybrid nanoparticles. All together, these results indicate that, in terms of biocompatibility and combined antibacterial plus tissue regenerative activities, the CS/Fe_3_O_4_ are the best hybrid nanoparticles synthesized by ionotropic gelation strategy. Therefore, this chitosan/iron nanocomposite can be considered as a very promising candidate for its biomedical applications in skin damage care.

## Figures and Tables

**Table 1:** Optimization study of ionotropic gelation-based synthesis of CS and CS-based hybrid NPs and their representative characterizations.

Sample	CS/TPP Ratio (*w*/*w*)	Core Size (nm)	Hydrodynamic Size (nm)	PDI *	ζ-Potential (mV) pH 7
**CS NPs**	2.4/1	33.7 ± 5.85	109.8	0.19	8.1
**Fe_3_O_4_ NPs**	-	8.18 ± 1.57	19.98	0.18	3,6
**CS/Fe_3_O_4_ NPs**	2.6/1	-	123.1	0.12	7.5
**Ag NPs**	-	33.02 ± 13.02	108.2	0.29	−11.8
**CS/Ag NPs**	2.5/1	-	121.8	0.22	7.7
**Au NPs**	-	14.05 ± 1.61	33.0	0.21	−17.4
**CS/Au NPs**	2.5/1	-	121.5	0.30	7.5

* PDI: Polydispersity Index.

**Table 2:** Yields of ionotropic gelation-based syntheses of CS and CS-based hybrid NPs and their chemical composition characterizations.

Sample	Inorganic NPs	Synthesis Yield (%)	Metal Encapsulation (%)	mg of Inorganic NPs in 1 mg/mL of Hybrid NPs	mg of CS in 1 mg of Hybrid NPs
**CS NPs**	-	65	-	-	1
**CS/Fe_3_O_4_ NPs**	Fe_3_O_4_	20	90.0	0.26	0.74
**CS/Ag NPs**	Ag	28	91.5	0.16	0.84
**CS/Au NPs**	Au	35	86.4	0.14	0.86

## Data Availability

Not applicable.

## Supplementary Materials

The following are available online at https://www.mdpi.com/article/10.3390/polym13223910/s1, Figure S1. TEM micrographs and size distribution (log-normal fit) of (a) iron oxide nanoparticles (IONPs), (b) silver nanoparticles (Ag NPs) and c) gold nanoparticles (Au NPs), Figure S2. TEM micrographs and size distribution (log-normal fit) of chitosan nanoparticles (CS NPs), Figure S3. Cytotoxicity assay of CS NPs in MEF cells, Figure S4. Wound healing assay of CS NPs and CS/metal hybrid NPs at 24 and 48 h.
