# Supplementary material for: Ionotropic Gelation-Based Synthesis of Chitosan-Metal Hybrid Nanoparticles Showing Combined Antimicrobial and Tissue Regenerative Activities
Source: Polymers (Basel). 2021 Nov 12;13(22):3910. doi: 10.3390/polym13223910 (PMC8618652; doi:10.3390/polym13223910)
Supplement: Supplementary file 1 [file polymers-13-03910-s001.zip › polymers-1433036-supplementary.pdf]

Supplementary Materials

Ionotropic gelation-based synthesis of chitosan-metal hybrid nanoparticles showing combined antimicrobial and tissue regenerative activities.

Laura Lozano Chamizo[1,2,3], Yurena Luengo Morato[1], Karina Ovejero Paredes[1,2], Rafael Contreras Caceres, Marco Filice[1,2*], Marzia Marciello[1*]

[1]*Nanobiotechnology for Life Sciences Lab., Department of Chemistry in Pharmaceutical Sciences, Faculty of Pharmacy, Universidad Complutense de Madrid (UCM), Plaza Ramón y Cajal s/n, E-28040 Madrid, Spain; laurloza@ucm.es (L.L.C.); yluengo@ucm.es (Y.L.M.); kovejero@ucm.es (K.O.P.); mfilice@ucm.es (M.F.); marmarci@ucm.es (M.M);*

[2]*Microscopy and Dynamic Imaging Unit, Fundación Centro Nacional de Investigaciones Cardiovasculares Carlos III (CNIC), Calle Melchor Fernández Almagro 3, E-28029 Madrid, Spain; laurloza@ucm.es (L.L.C.); kovejero@ucm.es (K.O.P.); mfilice@ucm.es (M.F.)*

[3]*Atrys Health, E-28001 Madrid, Spain; laurloza@ucm.es (L.L.C.)*

[4] *Department of Chemistry in Pharmaceutical Sciences, Faculty of Pharmacy, Universidad Complutense de Madrid (UCM), Plaza Ramón y Cajal s/n, E-28040 Madrid, Spain; rafcontr@ucm.es (R.C.C.)*

**Correspondence: marmarci@ucm.es (M.M); mfilice@ucm.es (M.F.)*

1. CS and hybrid CS-based nanoparticles preparation

CS and hybrid NPs were prepared using an ionotropic gelation technique. Various ratios between their CS, TPP and/or inorganic NPs constituents were studied to find the best relation for obtaining the highest NPs higher yield. In fact, sing an excess of CS, NPs were not formed. In turn, with an excess of TPP amount, the precipitation Cs NPs is observed. This behaviour is explained by the CS-based NPs formation mechanism [1]. CS is a polymer that shows amino groups that in acidic aqueous media are positively charged, thus, offering a large number of cross-linking sites. In these conditions a negative charged molecules, as TPP, can interact with these charged amino groups and generating an ionic cross-linked structure of CS. The presence of an excess of negative charges, led to precipitation, probably due to a supersaturation of CS crosslinking sites that led to an engagement of more CS molecules in a single NP formation. As a consequence, CS NPs with larger particle size and low surface charge density were formed, followed by precipitation [2]. On the other hand, if the cationic cross linking sites of CS are not sufficiently involved in the final network, CS NPs are not completely formed and the suspension appear transparent.

For this reason, an optimal CS/TPP ratio (w/w) is crucial to obtain small and monodisperse CS NPs showing an opalescent NPs suspension.

Even in the case of hybrid inorganic-CS NPs, the optimization of the ratio within the CS, TPP and/or inorganic NPs constituents is fundamental for a successful synthesis. The incorporation of inorganic NPs with a CS/ratio of 2.4 (w/w) was not successful. Due to a competition between TPP and negative charged inorganic NPs for the same CS positive sites, precipitation phenomenon was observed. To obtain the correct component ratio, TPP was gradually reduced until a CS/TT ratio of 2.5 (w/w) for Ag and Au hybrid NPs and 2.6/1 (w/w) in the case of Fe_3O_4 NPs.

2. Supplementary Figures

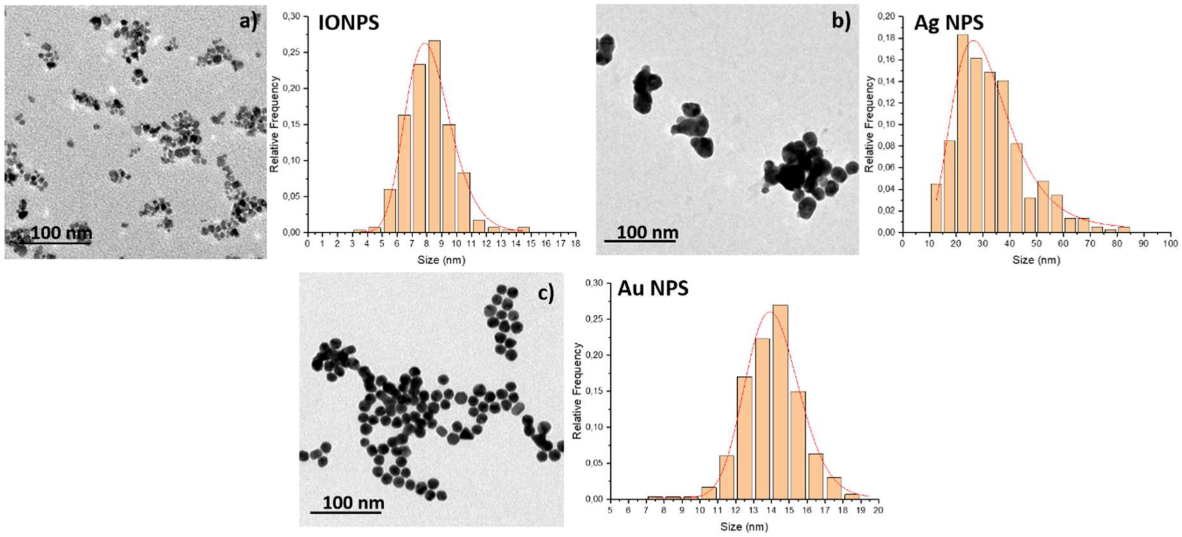

Figure S1. TEM micrographs and size distribution (log-normal fit) of a) iron oxide nanoparticles (IONPs), b) silver nanoparticles (Ag NPs) and c) gold nanoparticles (Au NPs).

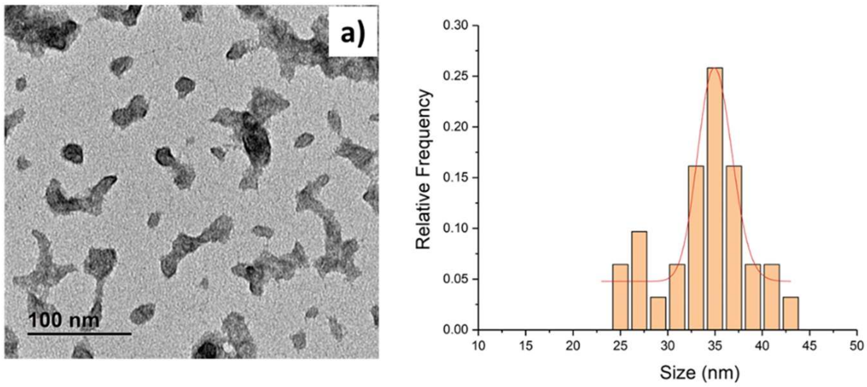

Figure S2. TEM micrographs and size distribution (log-normal fit) of chitosan nanoparticles (CS NPs).

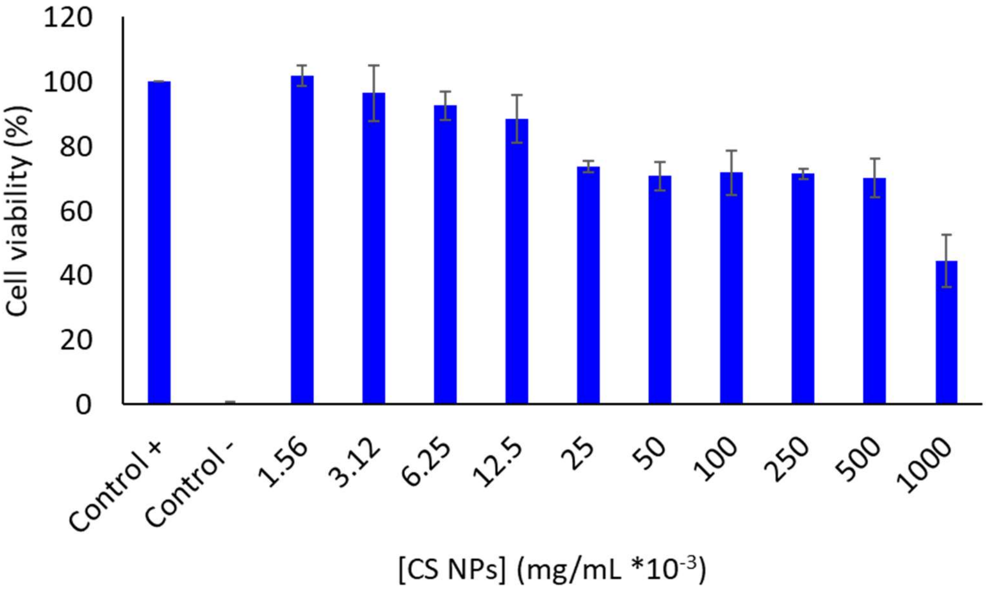

Figure S3. Cytotoxicity assay of CS NPs in MEF cells.

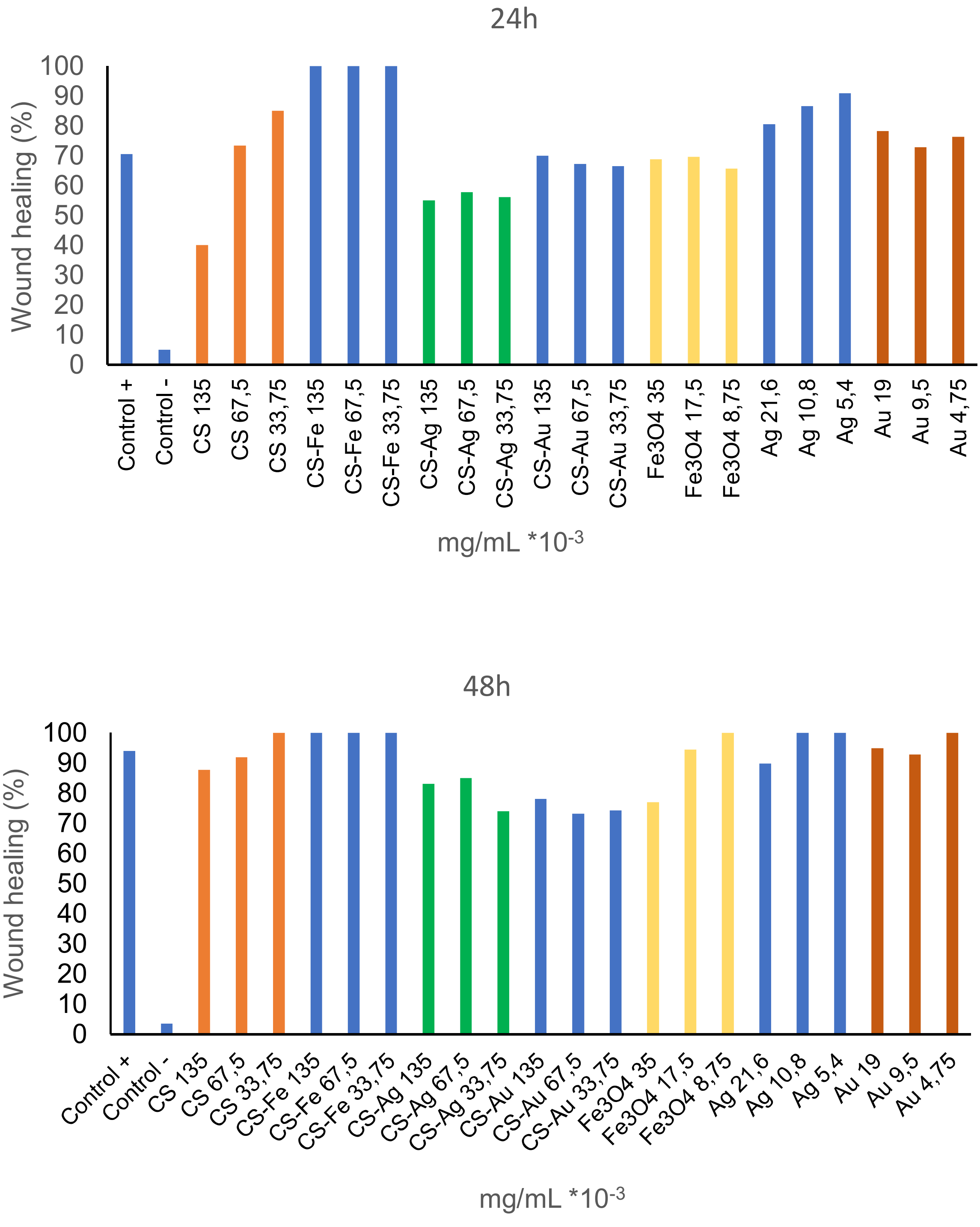

Figure S4. Wound healing assay of CS NPs and CS/ metal hybrid NPs at 24 and 48h.

References:

[1] M. Marciello, S. Rossi, C. Caramella, C. Remuñán-López, Freeze-dried cylinders carrying chitosan nanoparticles for vaginal peptide delivery, Carbohydrate Polymers 170 (2017) 43-51.
[2] W. Fan, W. Yan, Z. Xu, H. Ni, Formation mechanism of monodisperse, low molecular weight chitosan nanoparticles by ionic gelation technique, Colloids and Surfaces B: Biointerfaces 90 (2012) 21-27.
